# Monitoring Antigenic Variations of Enterovirus 71: Implications for Virus Surveillance and Vaccine Development

**DOI:** 10.1371/journal.pntd.0003044

**Published:** 2014-07-24

**Authors:** Min-Yuan Chia, Wan-Yu Chung, Pai-Shan Chiang, Yeh-Sheng Chien, Mei-Shang Ho, Min-Shi Lee

**Affiliations:** 1 National Institute of Infectious Diseases and Vaccinology, National Health Research Institutes, Zhunan, Taiwan; 2 Institute of Biomedical Sciences, Academia Sinica, Taipei, Taiwan; Aix Marseille University, Institute of Research for Development, and EHESP School of Public Health, France

## Abstract

Enterovirus 71 (EV71) causes life-threatening epidemics in Asia and can be phylogenetically classified into three major genogroups (A∼C) including 11 genotypes (A, B1∼B5, and C1∼C5). Recently, EV71 epidemics occurred cyclically in Taiwan with different genotypes. In recent years, human studies using post-infection sera obtained from children have detected antigenic variations among different EV71 strains. Therefore, surveillance of enterovirus 71 should include phylogenetic and antigenic analysis. Due to limitation of sera available from children with EV71 primary infection, suitable animal models should be developed to generate a panel of antisera for monitoring EV71 antigenic variations. Twelve reference strains representing the 11 EV71 genotypes were grown in rhabdomyosarcoma cells. Infectious EV71 particles were purified and collected to immunize rabbits. The rabbit antisera were then employed to measure neutralizing antibody titers against the 12 reference strains and 5 recent strains. Rabbits immunized with genogroup B and C viruses consistently have a lower neutralizing antibody titers against genogroup A (≧8-fold difference) and antigenic variations between genogroup B and C viruses can be detected but did not have a clear pattern, which are consistent with previous human studies. Comparison between human and rabbit neutralizing antibody profiles, the results showed that ≧8-fold difference in rabbit cross-reactive antibody ratios could be used to screen EV71 isolates for identifying potential antigenic variants. In conclusion, a rabbit model was developed to monitor antigenic variations of EV71, which are critical to select vaccine strains and predict epidemics.

## Author Summary

Enterovirus 71 (EV71) has caused several life-threatening epidemics in children in the Asia-Pacific region since 1997. EV71 has one single serotype as measured using hyper-immune animal antisera but can be phylogenetically classified into three major genogroups (A, B and C) and eleven genotypes (A, B1–B5, and C1–C5). Recently, epidemiological studies in the Asia-Pacific region have found that large-scale EV71 epidemics occurred cyclically with different genotypes. This observation of genotype replacement, in conjunction with the observed antigenic variations among different EV71 genogroups in human studies, has kindled the interest to establish animal models to monitor the antigenic variations of EV71. In this study, we developed a rabbit model to monitor antigenic variations of EV71, which could be further integrated into national enterovirus surveillance systems.

## Introduction

Enterovirus 71 (EV71) is a non-enveloped RNA virus of the family *Picornaviridae* and contains a positive sense ssRNA with a single open reading frame (ORF). The ORF is expressed as a large polyprotein that can be cleaved into P1, P2 and P3 regions. The P1 gene encodes four structural proteins VP1–VP4, while P2 and P3 genes encode the non-structural proteins responsible for virus replication and virulence [1]. The viral icosahedral capsid is composed of 60 identical units that consist of VP1–VP4 structural proteins [2], [3]. Variation of capsid proteins, except VP4, is responsible for the antigenic diversity among the enteroviruses, while neutralizing epitopes and phylogenetic classification are mainly based on VP1 and VP2 [4]–[7].

According to analysis of VP1 sequences, EV71 was phylogenetically divided into three distinct genogroup: A, B, and C [8], [9]. Genogroups B and C can be further divided into genotypes B1–B5 and C1–C5, respectively [10]. Recently, genogroup D was identified in India and genogroups E and F were identified in Africa [11], [12]. Genogroup A composes of the prototype EV71 strain (BrCr-CA-70) which was isolated in 1970 in the United States but had not been detected afterwards until 2008. In contrast, genogroup B and C viruses have been causing large scale of epidemics in Asia since 1997 and are targeted for vaccine development [10], [13].

Most EV71 infections manifest as mild cases of hand-foot-mouth disease (HFMD) or herpangina in young children, who are potentially at risk for severe neurological and cardiopulmonary complications [8], [9]. The neurovirulence of EV71 first came to people's attention in California in 1969 [14]. Since then, EV71 has caused several outbreaks sporadically in the 1970s, i.e. 1975 in Bulgaria, 1978 in Hungary [15], [16]. Since 1997, EV71 has been further identified as the causative agent responsible for the epidemics of central nervous system disease occurring in Asia-Pacific countries [9], [17]. In Taiwan, phylogenetic analyses revealed that different predominant genotypes occurred in 1998 (C2), 2000–2001 (B4), 2004–2005 (C4), and 2008 (B5) [10], [18]. This genotype replacement has also been observed in Malaysia and Vietnam [10], [19], [20]. Therefore, continuous monitoring genetic and antigenic evolution of EV71 are critical to vaccine development and epidemic control. Although EV71 has one single serotype as measured using hyper-immune animal sera, recent human studies using post-infection sera obtained from children to measure cross-neutralizing antibody titers against different genotypes have detected antigenic variations among different EV71 strains [21]–[24]. Due to the limitation of small amount of sera available from young children with EV71 primary infection, suitable animal models should be developed to generate a panel of antisera for monitoring EV71 antigenic variations.

In the present study, 12 reference viruses representing the eleven EV71 genotypes were collected and purified to immunize rabbits for generating EV71-specific rabbit antisera. Then, cross-reactive neutralizing antibody titers among the 12 reference viruses and sera were measured for evaluating antigenic changes. Finally, 5 recent circulating strains in Taiwan were analyzed genetically and antigenically using the 12 reference antisera.

## Materials and Methods

### Ethics statement

The animal protocol was approved by the NHRI Institutional Animal Care and Use Committee (approval no. NHRI-IACUC-100008-A) following the Institutional Animal Care and Use Committee Guidebook published by the US Office of Laboratory Animal Welfare (http://grants.nih.gov/grants/olaw/guidebook.pdf).

### Viruses and cells

**Figure 1:**
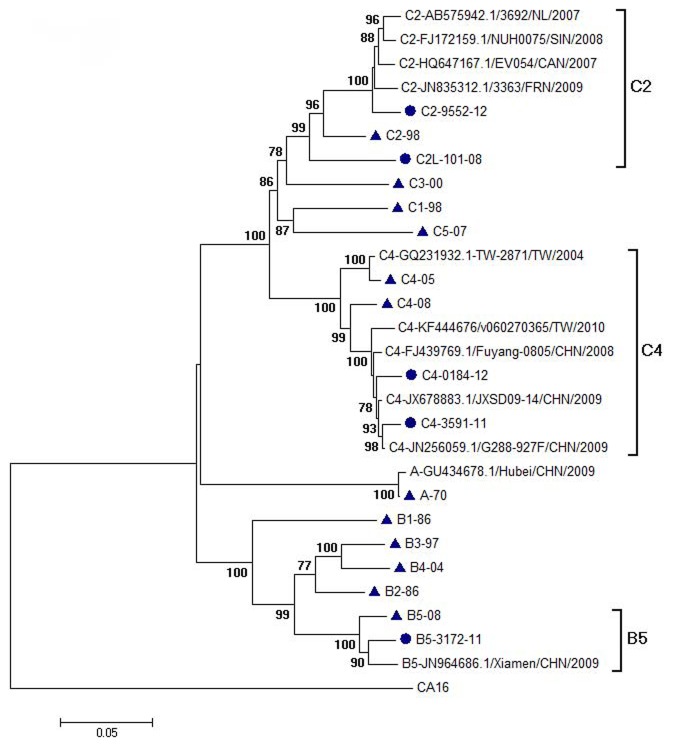
Phylogenetic analysis of EV71 strains. The phylogenetic tree was constructed using nucleotide sequences of P1 genes and the reliabilities indicated at the branch nodes were evaluated using 1,000 bootstrap replications. Only bootstrap values of over 70% were shown. The prototype coxsackievirus A16 (CA16) G-10 strain was used as an out-group. Solid circle : recent strains; Solid triangle : reference strains.

Twelve reference viruses representing 11 EV71 genotypes were collected and used in the present study. Eight of these viruses were isolated in Taiwan and the other four viruses (genotype A, B2, B3 and C3) were isolated in other countries (Table 1) [24]. Besides, five recent strains include 4 strains (one B5, one C2 and two C4) isolated in 2011 and 2012 and one C2-like virus (C2L-101-08) which was isolated in 2008 and identified as an antigenic variant using post-infection sera obtained from children in a previous study (Table 2) (Figure 1) [22]. Viral isolation methods had been described previously [22]. All viruses were amplified in rhabdomyosarcoma (RD) cells using Dulbecco's Minimum Essential Medium (DMEM) containing 2% v/v fetal bovine serum and penicillin/streptomycine. The 50% tissue culture infective dose (TCID_50_) of virus was calculated in RD cells using the Reed-Muench method.

**Table 1:** Twelve reference EV71 genotypes and five recent circulating strains used for measuring cross-neutralizing antibody titers in this study.

Genotype	Name	Year	Country	Abbreviate	Accession No.
Reference strains					
A	A-U22521-BrCr	1970	USA	A-70	JN874547
B1	242-TW-86	1986	Taiwan	B1-86	JN874548
B2	86-11316	1986	Netherlands	B2-86	JN874549
B3	SK-EV006	1997	Malaysian	B3-97	JN874550
B4	E59P2-TW-02	2002	Taiwan	B4-02	JN874551
B5	NHRI141-TW-08	2008	Taiwan	B5-08	JN874552
C1	TW-4215-1998	1998	Taiwan	C1-98	JN874553
C2	Tainan/5746/98	1998	Taiwan	C2-98	JN874554
C3	001-KOR-00	2000	Korea	C3-00	JN874555
C4	N1862-TW-05	2005	Taiwan	C4-05	JN874556
C4	70516TW-08	2008	Taiwan	C4-08	JN874557
C5	1575TW-07	2007	Taiwan	C5-07	JN874558
Recent strains					
B5	3172-TW	2011	Taiwan	B5-3172-11	KF306100
C2	NHRIEV9552	2012	Taiwan	C2-9552-12	KF306101
C2-Like	C2-L 1101/XL1	2008	Taiwan	C2L-101-08	HM622391
C4	3591-TW	2011	Taiwan	C4-3591-11	KF306099
C4	0184-TW	2012	Taiwan	C4-0184-12	KF306098

**Table 2:** Neutralizing antibody titers of rabbit antisera against twelve reference and five recent strains.

Reference strains	Rabbit antisera											
	A-70	B1-86	B2-86	B3-97	B4-02	B5-08	C1-98	C2-98	C3-00	C4-05	C4-08	C5-07
A-70	**256**	8	8	<8	<8	32	<8	8	<8	<8	16	8
B1-86	32	**512**	512	16	128	1024	256	64	512	128	32	256
B2-86	32	512	**2048**	8	128	2048	256	64	512	32	32	64
B3-97	32	32	256	**512**	64	512	128	32	128	16	512	16
B4-02	64	1024	2048	8	**1024**	4096	1024	256	1024	256	64	256
B5-08	32	128	1024	8	128	**4096**	512	64	128	128	64	64
C1-98	16	32	512	256	32	512	**512**	64	128	32	128	32
C2-98	16	32	1024	8	32	1024	1024	**512**	256	32	64	32
C3-00	32	1024	2048	8	256	4096	1024	64	**1024**	256	64	256
C4-05	128	1024	2048	8	512	4096	512	512	1024	**512**	256	512
C4-08	16	64	1024	64	16	512	512	32	128	64	**1024**	64
C5-07	32	512	1024	8	128	1024	1024	256	512	256	32	**256**
**Recent strains**												
B5-3172-11	16	32	256	64	64	512	256	32	64	32	128	64
C2-9552-12	32	16	128	64	16	512	512	16	64	16	2048	32
C2L-101-08	8	<8	<8	8	<8	8	<8	8	<8	<8	8	<8
C4-3591-11	32	64	1024	8	64	2048	1024	64	256	64	128	16
C4-0184-12	32	32	512	128	32	1024	1024	64	128	32	4096	32

### RT-PCR and sequencing

Total viral RNA was extracted from culture supernatant by QIAamp Viral RNA Kit (Qiagen) according to the manufacturer's instruction. The reverse transcription polymerase chain reaction (RT-PCR) and sequencing were performed as previously described [24]. The P1 regions (2586 bp) of the 12 reference and five recent circulating strains were aligned and analyzed using a *Clustal W* in *MEGA 4*. Identification and genotyping was carried out using phylogenetic analysis, which was conducted with 1,000 replications of bootstrap analyses using a Neighbor-Joining model and the prototype coxsackievirus A16 (CA16) G-10 strain was used as an outgroup virus [25].

### Purification of EV71

It is well known that two forms of EV71 viral particles, full and empty particles, existed during propagation in cells [3], [26]. Based on historical poliovirus studies, the full particles are infectious and immunogenic but the empty particles are not [27]. Therefore, we purified EV71 infectious (full) particle of the reference viruses for rabbit immunizations. The EV71 culture supernatant was concentrated 10-fold with a Amicon 100K centrifugal filter (Millipore). The crude virus concentrate was loaded onto a 15–65% continuous sucrose gradient and centrifuged at 28000 rpm for 4 hr. Fractions (2 mL per fraction) were collected and the viral titer and protein concentration of each fraction were determined by TCID_50_ and BCA assays (Thermo Scientific), respectively. Fractions with high infectious virus titers in 32–38% sucrose concentration were merged and concentrated by diafiltration using Amicon 100K centrifugal filter and centrifugation at 3500 g. The purified EV71 viruses were further verified by using Western blot and electron microscopy analysis.

### Transmission electron microscopy analysis

Purified EV71 particles were deposited on a carbon-coated 200 mesh copper grid for 1 min at room temperature. The excess sample was removed by filter paper and then the copper grid was stained with 2% phosphotungstic acid solution for 1 min, which was then removed by filter paper. The stained grid was dried for 1 day at room temperature and observed under a JEM 1200EX transmission electron microscopy [26].

### Preparation of rabbit antisera

Two-month-old New Zealand White rabbits were immunized three times subcutaneously with purified infectious EV71 viruses (1×10^7^ TCID_50_ in 1 mL PBS). The immunized rabbits were bled one week after the final boost, and the sera were collected and stored at −20°C for further analysis. The animal protocol was approved by the NHRI IACUC.

### Determination of neutralizing antibody titers

Serum neutralizing antibody titers were detected using TCID_50_ assay according to the standard protocol [28]. Serum samples from immunized rabbits were inactivated for 30 min at 56°C, and then diluted two-fold serially in DMEM with an initial dilution of 1∶8. Fifty µl of diluted sera and 100 TCID_50_ viruses were added to 96-well microplates and incubated at 37°C for 1 hr. Later, 100 µl of RD cell suspension containing 3×10^4^ cells was added, and cytopathic effect (CPE) was observed in the inverted microscopic after an incubation at 37°C for 3–4 days. The neutralization titers were defined as the highest dilution that could result in a >50% reduction in the CPE. Each test sample was run simultaneously with positive serum control, cell control and virus back-titration.

### Antigenic cartography

Antigenic cartography is a way to visualize and increase the resolution of serological data [21], [24]. In an antigenic map, the distance between a serum point S and antigen point A corresponds to the difference between the log_2_ of the maximum titer observed for serum S against any antigen and the log_2_ of the titer for serum S and antigen A. Thus, each titer in a neutralization assay can be thought of as specifying a target distance for the points in an antigenic map. In this study, an antigenic map was generated using a web-based analytic tool [29].

### Locating amino acid positions in the 3-D structure of EV71 capsid protein

Recently, 3-dimensional (3-D) structures of P1 protein of two EV71 virus strains (genotype B3 and genotype C4) were generated using X-ray crystallography (PDB 4AED and PDB 3VBS) [2], [3]. The 3-D structure of the genotype C4 virus was based on infectious particles so it was employed as template to locate specific amino acid positions using the RasMol software (http://rasmol.org/).

### Comparison between rabbit and human cross-reactive neutralizing antibody profiles

To compare human and rabbit cross-reactive neutralizing antibody profiles, cross-reactive antibody ratios between homotypic and heterotypic antibody titers were calculated because measurement of neutralizing antibody titers tends to be highly variable and cross-reactive antibody ratios won't be affected by absolute antibody titers. In our previous study, 21 sera collected from children infected with EV71 genotype C2-1998 (6 sera), C4-2005 (2 sera), C4-2010 (3 sera), B4-2002 (5 sera) and B5-2008 (5 sera) viruses were available to measure cross-reactive neutralizing antibody titers against all 12 reference viruses [24]. Serum antibody titers of children infected with EV71 in the same year were merged to calculate geometric mean titers. Therefore, 55 cross-reactive antibody ratios between homotypic and heterotypic antibody titers were available from human serology data. For rabbit serology data, there were 132 cross-reactive antibody ratios among 12 reference viruses. After combining human and rabbit serology data, 55 cross-reactive antibody ratios were available to evaluate the correlation between human and rabbit cross-reactive antibody profiles.

### Statistical analysis

Differences between homologous and heterologous neutralizing antibody titers were tested for statistical significance by using Student's *t-*test with log2-transformed data. The *P* value<*0.05* is taken to indicate statistically significance.

## Results

### Purification of EV71 infectious particles

**Figure 2:**
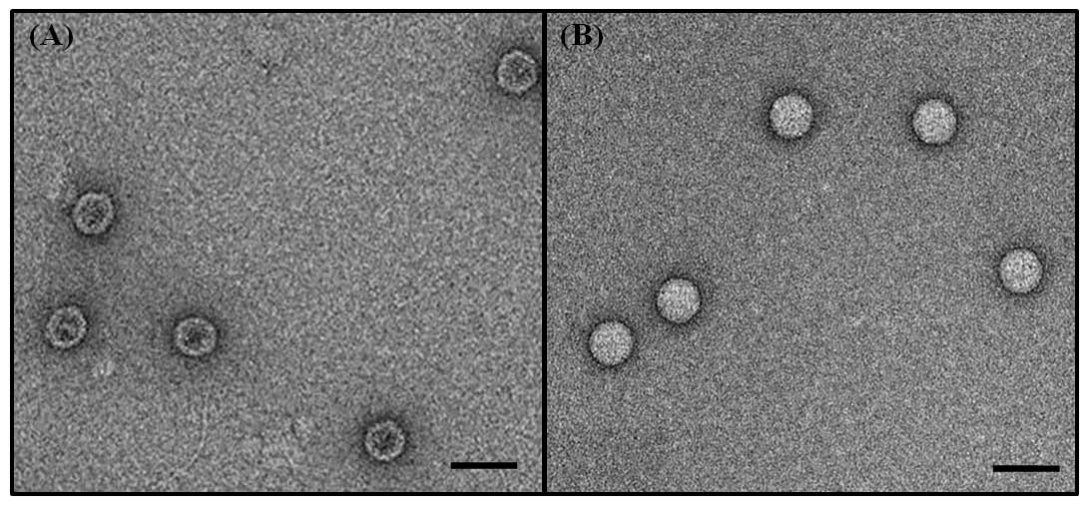
Photographs of EV71 viral particles analyzed by transmission electron microscopy. (A) The empty particles had a defective structure with approximately 30–32 nm in diameter. (B) The full particles had a solid structure with 33–35 nm in diameter. The bar indicates 50 nm.

Twelve EV71 reference viruses representing 11 EV71 genotypes were collected (Table 1) and their P1 genes were sequenced to confirm their genotypes using phylogenetic analysis (Figure 1). Two genotype C4 viruses were selected due to high diversity existing in this genotype. These 12 reference viruses were amplified in RD cells and purified by sucrose gradient ultracentrifugation. After ultracentrifugation, all fractions of sucrose gradients were collected for quantification of sucrose concentration, measurement of infectious virus titers and detection of viral proteins using Western blot analysis. The fractions with the highest virus titers usually located at fractions with 32–38% sucrose and they were merged for further analysis (F32–38) and used to immunize rabbits for generating antisera. In addition, several fractions with low virus titers but high concentration of viral proteins usually located at fractions with 24–30% sucrose and they were also merged for further analysis (F24–30). In electron microscopy analysis, the viruses were purified by second 15–65% continuous sucrose gradient ultracentrifugation and empty particles (F24–30) and full particles (F32–38) were observed in negative staining (Figure 2). Some previous studies had demonstrated that two different types (full and empty types) EV71 particles were separated by using sucrose gradient ultracentrifugation and contained different protein conformations [3], [26].

### Cross-reactive neutralizing antibody profiles

**Figure 3:**
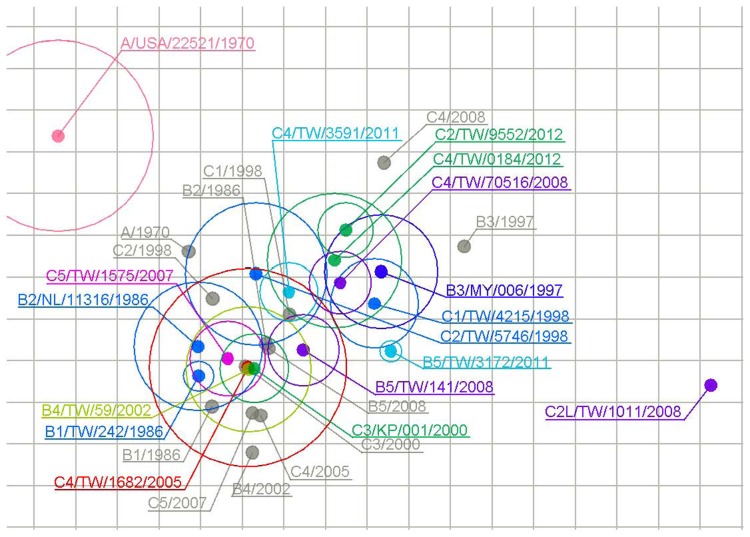
Antigenic map of twelve rabbit antisera against 11 representative genotypes and 5 recent circulating strains. The relative positions of viral strains (color points) and antisera (grey points) were adjusted such that the distances between strains and antisera in the map represent the corresponding neutralization titers. Each strain has a outer circle which points out the range of standard error. The space between grid lines is 1 unit of antigenic distance, corresponding to a 2-fold dilution of antiserum in the neutralization assay.

To generate rabbit antisera for determining cross-reactive neutralizing antibody profiles among EV71 genotypes, rabbits were immunized with the full particles of the reference viruses and these antisera were collected for measuring cross-reactive neutralizing antibody titers against 12 reference strains and 5 recent strains (Table 2). The 5 recent strains included 4 strains (one B5, one C2 and two C4) isolated in 2011 and 2012 and one C2-like virus (C2L-101-08) which was isolated in 2008 and identified as an antigenic variant using post-infection sera obtained from children in a previous study (Table 2) (Figure 1) [22]. As shown in Table 2, the 12 reference EV71 viruses induced high homotypic neutralization titers (1∶256 to 1∶4096). Regarding to cross-reactive antibody responses, genotype A virus (A-70) consistently has >8-fold difference between homotypic and heterotypic neutralizing antibody titers but no clear pattern could be identified for genogroup B and C viruses. Interestingly, genotype B2 and B5 viruses seems to be highly immunogenic and could induce high homotypic and heterotypic neutralizing antibody titers against all genogroup B and C viruses except the C2-like virus isolated in 2008. Among the 5 recent strains, the C2-like virus is an antigenic outlier and the other 4 recent viruses were antigenically similar to their homotypic reference viruses. To better visualize the cross-reactive serological data, antigenic cartography was further employed to analyze the serological data and showed that genotype A virus and the C2-like virus could be antigenically differentiated from other EV71 viruses (Figure 3), which are consistent to previous human studies.

### Comparison between human and rabbit neutralizing antibody profiles

**Figure 4:**
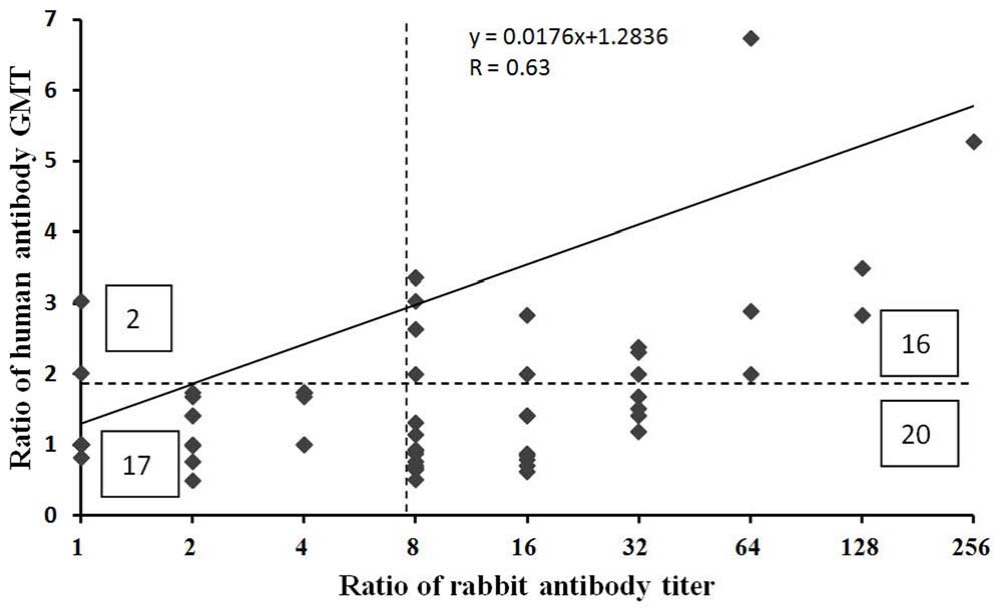
Scatter plot between ratios of homotypic and heterotypic GMTs in humans and cross-reactive antibody titers in rabbits. Based on influenza experience, ≧50% differences in GMT of cross-reactive antibody titers in humans (i.e. ≧2 in cross-reactive antibody ratios) may cause decreased vaccine efficacy [30]. The horizontal and vertical dashed lines indicate cut-off of 2 in ratio of human GMTs and cut-off of 8 in ratio of cross-reactive antibody titers in rabbits. Numbers in each corner indicate true positive (upper right), false positive (lower right), false negative (upper left), and true negative (lower left).

In our previous study, 21 sera collected from children infected with EV71 genotype C2-1998 (6 sera), C4-2005 (2 sera), C4-2010 (3 sera), B4-2002 (5 sera) and B5-2008 (5 sera) viruses were available to measure cross-reactive neutralizing antibody titers against all 12 reference viruses [24]. Serum antibody titers of children infected with the same EV71 genotype were merged to calculate geometric mean titers (GMTs). Therefore, 55 cross-reactive antibody ratios between homotypic and heterotypic GMTs were available from human serology data. For rabbit serology data, there were 132 pair-wise cross-reactive antibody ratios among the 12 reference viruses. After combining human and rabbit serology data, 55 cross-reactive antibody ratios were available to evaluate the correlation between human and rabbit cross-reactive antibody profiles. Scatter plot between human and rabbit cross-reactive antibody ratios are shown in Figure 4, which shows that cross-reactive antibody ratios ranged from 1 to 7 in human data and from 1 to 256 in rabbit data. Overall, the cross-reactive antibody ratios calculated using human and rabbit serology data correlate to each other (correlation coefficient R = 0.63, *P<0.01*) (Figure 4). Based on influenza studies, ≧50% differences in GMT of cross-reactive antibody titers in humans (i.e. ≧2 in cross-reactive antibody ratios) may cause decreased vaccine efficacy [30]. Using the same criteria for human EV71 serology data, 18 cross-reactive antibody ratios were ≧2 and 16 of them (89%) could be identified by using ≧8-fold differences in rabbit cross-reactive antibody ratios. Therefore, ≧8-fold difference in rabbit cross-reactive antibody ratios could be used to screen EV71 isolates for identifying potential antigenic variants. Then, the potential EV71 antigenic variants identified using rabbit antisera would be further verified using post-infection sera obtained from children.

### Identifying amino acid positions related to antigenic variations

**Figure 5:**
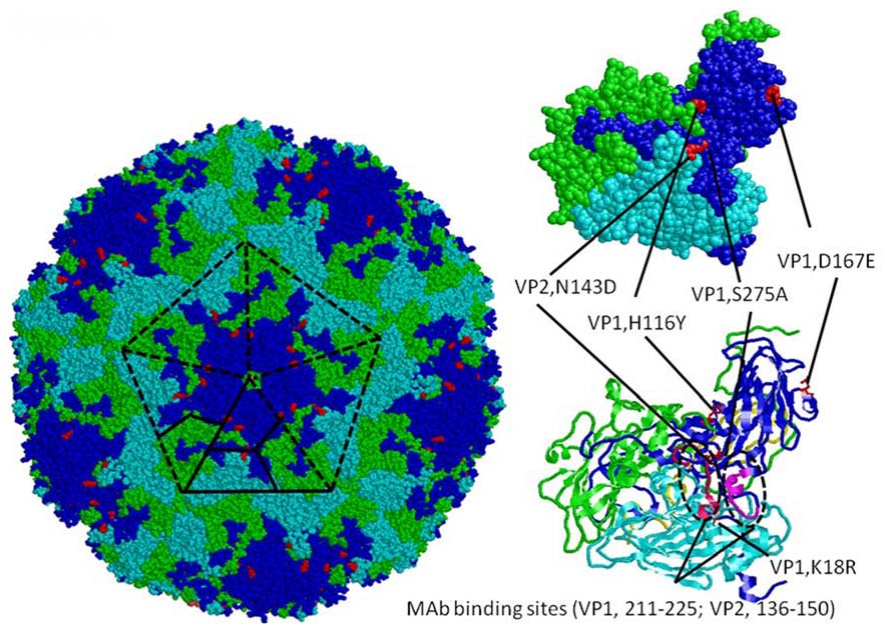
Five amino acid mutations which may be related to antigenic variation of EV71 genotype A virus are located on 3-D structure of capsid protein (PDB 3VBS) using the RasMol software ( http://rasmol.org/ **).** VP1 (blue), VP2 (cyan), VP3 (green) and VP4 (yellow) proteins are presented in whole virus particle (left panel), space fill model of P1 polyprotein (upper right panel), and cartoon model of P1 polyprotein (lower right panel). Two neutralizing epitopes (VP1: 211–225; VP2: 136–151) identified using mouse monoclonal antibodies are shown in the cartoon model of P1 polyprotein.

Since two antigenic variants (A-70 and C2L-101-08) were identified, we further tried to identify amino acid positions related to the observed antigenic variations. As shown in Table 3, amino acid diversity rates were 0%, 8% (21/254), 3% (8/242), and 8% (25/297) for VP4, VP2, VP3 and VP1 regions, respectively. Five amino acid residues (VP2-143N, VP1-18K, VP1-116H, VP1-167D, and VP1-275S) are specific signatures for A-70 virus but no amino acid residue is specific for C2L-101-08 virus (Table 3). We further located these 5 residues in the 3-D structure of P1 polyprotein. As shown in Figure 5, 4 of the 5 residues are located on the surface and 3 (VP1-116, VP1-275 and VP2-143) of them are located in or near to neutralizing epitopes previously identified using mouse monoclonal antibodies (VP1 211-225 and VP2 136-150) [7]. Although no clear pattern could be identified by analyzing cross-reactive antibody profiles between genogroup B and C viruses, six amino acid residues (VP2-45D, VP2-126I, VP3-234H, VP3-240S, VP1-43E and VP1-58T) and seven amino acid residues (VP2-45S, VP2-198I, VP2-200A, VP3-100L, VP3-232A, VP3-239G and VP1-240T) were identified to be specific signatures for genogroup B and C viruses, respectively (Table 3).

**Table 3:** Alignment of amino acid sequences of P1 proteins of twelve reference and 5 recent strains.

	VP2 (aa 70–323)																					VP3 (aa 324–565)								VP1 (aa 566–862)																								
			1	1	2	2	2	2	2	2	2	2	2	2	2	2	2	2	2	2	3	3	4	4	5	5	5	5	5	5	5	5	5	5	6	6	6	6	6	7	7	7	7	7	7	8	8	8	8	8	8	8	8	8
From VP4	7	9	1	9	0	1	1	1	2	4	4	4	5	6	6	7	7	8	8	9	0	2	1	2	1	5	5	6	6	7	8	8	8	9	0	1	2	6	8	0	1	2	3	4	8	0	0	1	2	4	4	5	5	6
	2	5	4	5	7	0	2	3	5	4	6	8	1	7	9	0	4	7	8	3	7	9	6	3	7	5	7	2	3	9	1	3	7	6	8	7	3	3	1	8	0	9	2	9	5	5	6	4	7	0	5	4	7	2
				1	1	1	1	1	1	1	1	1	1	1	2	2	2	2	2	2	2			1	1	2	2	2	2										1	1	1	1	1	1	2	2	2	2	2	2	2	2	2	2
		2	4	2	3	4	4	4	5	7	7	7	8	9	0	0	0	1	1	2	3		9	0	9	3	3	3	4	1	1	1	2	3	4	5	5	9	1	4	4	6	6	8	2	4	4	4	6	7	8	8	9	9
	3	6	5	6	8	1	3	4	6	5	7	9	2	8	0	1	5	8	9	4	8	6	3	0	4	2	4	9	0	4	6	8	2	1	3	2	8	8	6	3	5	4	7	4	0	0	1	9	2	5	0	9	2	7
A-70	S	Q	N	V	G	T	N	S	D	L	V	P	W	M	T	L	S	P	I	F	L	L	D	M	V	T	D	A	T	D	V	K	Q	N	K	I	A	K	H	T	E	D	D	S	L	S	S	I	I	S	A	T	T	L
B1-86	T	.	D	I	V	S	D	.	.	R	.	.	.	.	.	.	.	.	.	.	.	.	.	.	.	.	H	.	S	.	M	R	.	.	E	.	T	.	Y	S	.	.	E	T	.	.	.	.	.	A		.	N	.
B2-86	.	.	D	I	.	.	D	.	.	.	.	H	R	.	.	.	.	.	.	.	.	.	.	.	.	.	H	.	S	.	.	R	.	.	E	.	T	E	Y	.	Q	E	E	T	.	.	.	V	.	A		.	S	.
B3-97	.	.	D	I	.	.	D	.	.	.	.	.	.	.	.	.	.	.	.	.	.	P	.	.	.	.	H	.	S	.	.	R	.	.	E	.	T	E	Y	.	G	E	E	T	.	.	L	V	.	A		.	.	.
B4-04	.	.	D	I	.	.	D	.	.	.	.	.	.	.	.	.	.	.	.	.	I	P	.	.	I	.	H	.	S	N	.	R	.	.	E	V	T	E	Y	.	Q	E	E	T	.	.	L	V	.	A		.	.	.
B5-08	.	.	D	I	.	.	D	.	.	.	I	.	.	.	.	.	.	.	.	.	.	P	.	.	.	.	H	.	S	.	.	R	.	.	E	.	T	E	Y	.	Q	.	E	T	.	.	.	V	.	A		.	.	.
C1-98	.	L	S	.	.	.	D	.	.	.	.	.	.	I	A	.	.	H	.	.	.	.	N	L	.	A	.	G	.	.	.	R	.	.	.	.	.	E	Y	.	Q	.	E	T	.	T	.	.	V	A		A	.	F
C2-98	.	.	S	.	.	.	D	.	.	.	.	.	.	I	A	.	.	.	.	Y	.	.	S	L	.	A	.	G	.	.	.	R	R	D	.	.	.	E	Y	.	.	.	E	.	.	T	.	.	.	A		A	.	.
C3-00	.	.	S	.	.	.	D	.	.	.	.	.	.	I	A	.	.	.	.	.	.	.	S	L	.	A	.	G	.	.	.	R	.	.	.	.	.	E	Y	.	Q	.	E	.	.	T	.	.	V	A		A	.	.
C4-05			S				D							I	A					Y			N	L		A		G				R							Y				E			T		V		A	G			
C4-08	.	.	S	.	.	.	D	.	.	.	.	.	.	I	A	.	.	.	.	Y	.	.	N	L	.	A	.	G	.	.	.	R	H	.	.	.	.	E	Y	.	.	.	E	.	I	T	.	V	.	A		.	.	.
C5-07	.	.	S	.	.	.	D	.	E	.	.	.	.	I	A	C	F	.	.	.	.	.	S	L	.	A	.	G	.	.	.	R	.	.	.	.	.	E	Y	.	.	.	E	.	.	T	.	.	V	A		A	.	.
B5-3172-11	.	.	D	I	.	.	D	.	.	.	I	.	.	.	.	.	.	.	.	.	.	P	.	.	.	.	H	.	S	.	.	R	.	.	E	.	T	E	Y	.	.	.	E	T	.	.	.	V	.	A		.	.	.
C2-9552-12	.	.	S	.	.	.	D	.	.	.	.	.	.	I	A	.	.	.	.	Y	.	.	S	L	.	A	.	G	.	.	.	R	R	.	.	.	.	E	Y	.	.	.	E	.	.	T	.	.	.	A		A	.	.
C2L-101-08	.	.	S	.	.	.	D	.	.	.	.	.	.	I	A	.	.	.	.	Y	.	.	S	L	.	A	.	G	.	.	.	R	.	.	.	.	.	E	Y	.	.	.	E	.	.	T	.	.	.	A		A	.	.
C4-3591-11	.	.	S	.	.	.	D	T	.	.	.	.	.	I	A	.	.	.	V	Y	.	.	N	L	.	A	.	G	.	.	.	R	H	.	.	.	.	E	Y	.	Q	.	E	.	.	T	.	V	.	A		A	.	.
C4-0184-12	.	.	S	.	.	.	D	T	.	.	.	.	.	I	A	.	.	.	.	Y	.	.	N	L	.	A	.	G	.	.	.	R	H	.	.	.	.	E	Y	.	.	N	E	.	.	T	.	V	.	A		A	.	.

The P1 protein consists of VP4 (aa 1–69), VP2 (aa 70–323), VP3 (aa 324–565) and VP1 (aa 566–862). Only amino acid residues with mutation were listed. Dots indicate amino acids identical to that of A-70 strain.

## Discussion

Although EV71 has one single serotype as measured using hyper-immune animal antisera, antigenic variations of EV71 have been identified in several human studies using post-infection sera obtained from children [22]–[24], [31]. Since it is hard to collect large amount of children sera for measuring cross-reactive neutralizing antibody titers, it would be desirable to establish an animal model for monitoring EV71 antigenic variations. In this study, a virus purification platform was established to purify EV71 infectious virus particles which were then used to generate rabbit antisera for measuring cross-reactive neutralizing antibody titers against reference and recent EV71 strains. The cross-reactive neutralizing antibody profiles defined using rabbit antisera were similar to those observed using post-infection sera obtained from children [23], [24], [32]. Moreover, ≧8-fold differences of cross-reactive antibody titers measured using rabbit antisera could be used as a screening criterion to select EV71 isolates for further evaluation using post-infection sera obtained from children, similar to ferret antisera used for global influenza surveillance [33], [34].

Two types (empty and full) of EV71 particles were produced in cell cultures and they could be separated using sucrose gradient ultracentrifugation in our study, which are similar to those observed in previous EV71 and poliovirus studies [3], [26], [35]. Based on historical poliovirus studies, full particles but not empty particles are highly immunogenic to induce neutralizing antibody responses so vaccine potency assay is based on the quantification of full particles [36]. Therefore, we used the EV71 full particles to generate rabbit antisera for monitoring antigenic variations.

Based on the cross-reactive neutralizing antibody profiles measured using the rabbit antisera, we identified two antigenic outliers (A-70 and C2L-101-08). The genotype A virus was first isolated in California in 1970 and disappeared for 38 years until 2008. Although genotype C4 viruses have been the predominant circulating viruses for the last 7 years in China [10], genotype A viruses have been sporadically detected in central (Anhui and Hubei), northern (Beijing) and western (Yunnan) China since 2008, which indicates widespread of genotype A viruses in China [13], [37]. Currently, genotype C4 and B4 viruses were used to develop EV71 vaccines in China and Taiwan, respectively and it would be critical to collect post-vaccination sera obtained from children to measure cross-reactive neutralizing antibody titers against the circulating genotype A viruses. Moreover, mechanism of the reemergence of genotype A viruses in China is not clear and could be clarified through genomics studies.

The novel C2-Like virus (C2L-101-08) was isolated in 2008, Taiwan. Children infected with genotype B4, B5, C4, and C5 viruses showed a maximum of 128-fold decrease in cross-reactive neutralizing antibody titers against C2L-101-08 compared with those of homogenous viruses. In the present study, all 12 rabbit antisera displayed low cross-reactive neutralizing antibodies (≦8-fold) against C2L-101-08 strain (Table 2) and the results were similar with post-infection children serological data [38]. It is indicated that C2L-101-08 strain exhibited evident antigenic diversity from other genotypes. Interestingly, the C2-like viruses were a minor group (2 strains/989 strains) in 2008 in Taiwan and have never been detected globally since 2009 to 2013 [22], [38], which indicate that the C2-like viruses do not have evolutionary advantages or become highly successful viral strain causing no/mild symptoms in humans. Surprisingly, no amino acid signature in the C2-like virus could be identified to be related to the observed antigenic variations. Several previous studies had demonstrated that some EV71 viruses were aggregated strains and they were poorly neutralized by anit-EV71 serum in a common neutralizing assay condition [39], [40]. In the present study, C2-like virus was treated with trypsin or chloroform for deaggregation but it still cloud not be neutralized by all 12 rabbit antisera. In addition, we tried to use the C2-like virus to generate rabbit antisera but did not succeed. Comprehensive genomic analysis and reverse genetics would be required to identify molecular determinants of the observed antigenic variations between the C2-like viruses and other EV71 viruses [41], [42].

In addition to the C2-like virus, four recent circulating strains including one genotype B5 (B5-3172-11), one genotype C2 (C2-9552-12), and two genotype C4 (C4-3591-11 and C4-0184-12) viruses isolated in Taiwan were also evaluated using the rabbit antisera in this study. The B5-3172-11 strain caused nation-wide epidemic in 2012 and was likely imported from Xiamen, China in 2011. In addition, the two recent genotype C4 viruses were also closely related to the C4 viruses circulating in China (Figure 1). Based on enterovirus surveillance in northern Taiwan, no genotype C2 virus was isolated in 2009–2011 and the C2-9552-12 virus was the only C2 strain isolated in 2012 and is phylogenetically related to the C2 strains isolated in Canada, France, Netherland, and Singapore in recent years (Figure 1). Based on rabbit cross-reactive neutralizing antibody profiles, the recent C2, C4 and B5 viruses did not antigenically significantly differ from their homotypic viruses. Interestingly, the C4 viruses were sporadically detected in 2010∼2011 in Taiwan but the B5-3172-11-like viruses were first detected in late 2011 and caused nation-wide epidemics in early 2012. It is likely the B5-2011 viruses had evolutionary advantages in replication and transmission efficiency in humans but did not cause antigenic drifts. Suitable EV71 animal transmission models would be desirable to elucidate the mechanism.

In the antigenic analysis using the 12 reference viruses and antisera, B2-86, B3-97, B4-02 and B5-08 viruses are in the same genogroup and have similar P1 sequences (Table 3). However, the B3-97 antisera could not efficiently neutralize the B2-86, B4-02 and B5-08 viruses (Table 2). Interestingly, the B2-86, B4-02 and B5-08 antisera could well neutralize the B3-97 virus. Comparing amino acid sequences of genogroup B in Table 3, one amino acid residue (VP1-145G) is the specific signature for B3-97 virus and it may be related to the observed antigenic variation, which is consistent to findings of other studies [38], [41].

In a recent study, a genotype C4 virus was used as an immunogen to generate 186 monoclonal antibodies (MAbs) and the MAbs with high neutralizing antibody titers were purified for antigenic analysis of eighteen EV71 clinical isolates [31]. The results showed that even EV71 strains in the same genotype do not generally produce similar antigenic profiles. These results indicate that the current genotyping of EV71 did not reflect their antigenicity, which is consistent with our study using rabbit antisera. However, the mouse monoclonal antibody profiles were not compared with human data. In contrast to the mouse monoclonal antibody study, our study showed that rabbit cross-reactive neutralizing antibody profiles are similar to the profiles measured using post-infection sera obtained from children. Since human antibody cross-reactive antibody profiles were polyclonal responses, it would be more suitable to use rabbit antisera other than mouse monoclonal antibody to monitor EV71 antigenic variations.

Due to significant impacts in public health, five organizations in Asia are developing EV71 vaccines using vaccine strains representing B2, B4, and C4 genotypes [10]. As shown in the present study and several previous studies, significant antigenic variations could be detected among different EV71 strains, especially the genotype A virus [10], [24], [43]. Currently, antigenic analysis would not be regularly conducted in the enterovirus 71 surveillance system. The rabbit model developed in the present study could be readily integrated into the national enterovirus surveillance system to monitor EV71 antigenic variations.
